# Migration, sexual behaviour, and HIV risk: a general population cohort in rural South Africa

**DOI:** 10.1016/S2352-3018(15)00045-4

**Published:** 2015-04-16

**Authors:** Nuala McGrath, Jeffrey W Eaton, Marie-Louise Newell, Victoria Hosegood

**Affiliations:** a Academic Unit of Primary Care and Population Sciences, University of Southampton, Southampton, UK; b Department of Social Statistics and Demography, University of Southampton, Southampton, UK; c Academic Unit of Human Development and Health, University of Southampton, Southampton, UK; d Africa Centre for Health and Population Studies, University of KwaZulu-Natal, Somkhele, South Africa; e Department of Infectious Disease Epidemiology, Imperial College London, London, UK

## Abstract

**Background:**

Increased sexual risk behaviour and HIV prevalence have been reported in migrants compared with non-migrants in sub-Saharan Africa. We investigated the association of residential and migration patterns with sexual HIV risk behaviours and HIV prevalence in an open, general population cohort in rural KwaZulu-Natal, South Africa.

**Methods:**

In a mainly rural demographic surveillance area in northern KwaZulu-Natal, South Africa, we collected longitudinal demographic, migration, sexual behaviour, and HIV status data through household surveillance twice per year and individual surveillance once per year. All resident household members and a sample of non-resident household members (stratified by sex and migration patterns) were eligible for participation. Participants reported sexual risk behaviours, including data for multiple, concurrent, and casual sexual partners and condom use, and gave a dried blood spot sample via fingerprick for HIV testing. We investigated population-level differences in sexual HIV risk behaviours and HIV prevalence with respect to migration indicators using logistic regression models.

**Findings:**

Between Jan 1, 2005, and Dec 31, 2011, the total eligible population at each surveillance round ranged between 21 129 and 22 726 women (aged 17–49 years) and between 20 399 and 22 100 men (aged 17–54 years). The number of eligible residents in any round ranged from 24 395 to 26 664 and the number of eligible non-residents ranged from 17 002 to 18 891 between rounds. The stratified sample of non-residents included between 2350 and 3366 individuals each year. Sexual risk behaviours were significantly more common in non-residents than in residents for both men and women. Estimated differences in sexual risk behaviours, but not HIV prevalence, varied between the migration indicators: recent migration, mobility, and migration type. HIV prevalence was significantly increased in current residents with a recent history of migration compared with other residents in the study area in men (adjusted odds ratio 1·19, 95% CI 1·07–1·33) and in women (1·18, 1·10–1·26).

**Interpretation:**

Local information about migrants and highly mobile individuals could help to target intervention strategies that are based on the identification of transmission hotspots.

**Funding:**

Wellcome Trust.

## Introduction

Increased sexual risk behaviour and high HIV prevalence in migrants compared with non-migrants in sub-Saharan Africa have led to research and prevention efforts that focus on migration as an individual risk factor and an important driver of HIV transmission.^1,2^ Additionally, studies have sought to estimate the effect of migration on population-level HIV prevalence.^3,4^ Common interpretations of the role of migration are centred around increased prevalence of sexual risk behaviours in migrants,^5,6^ and increased risk of HIV acquisition when destination communities have higher HIV prevalence than do origin communities.^2,7^ Differences in definitions and designs between studies present challenges in interpretation and comparison of empirical studies of migration and HIV risk, with some studies reporting no differences in HIV or sexual risk behaviours between migrants and non-migrants.^8,9^

The detailed demographic, migration, and HIV data available in several population-based cohorts and demographic surveillance systems have provided analysts with a valuable source of data for HIV risk studies.^10^ However, with a few exceptions, HIV surveys in these study populations have been restricted to adults who are residents in the study area.^11–13^

One exception is data available from the Africa Centre for Health and Population Studies Demographic Surveillance System (ACDIS) in rural KwaZulu-Natal, South Africa. In view of the very high levels of circular migration, in which adults migrate back and forth between rural and urban areas or other centres of employment,^14^ the ACDIS study population includes, and follows longitudinally, resident and non-resident members of households in the study area.^15^ Non-resident members are those deemed to belong to households in the rural areas despite being resident with another household either in the study area or outside. At each data collection round, information is recorded about new migrations of individuals and households into and out of the study area, and demographic data continues to be collected about non-resident members of households who are living elsewhere. Since 2003, ACDIS has administered HIV and sexual behaviour surveys every year, aiming to contact all adult residents. Between 2003 and 2011, the survey of individuals also included a sample of non-residents.^16^

**Research in context**

**Evidence before this study**

Before public HIV treatment programmes in sub-Saharan Africa, migration was widely understood to be positively associated with increased sexual risk behaviours and consequently with increased HIV prevalence in migrants and their partners. However, evidence for differential HIV prevalence in migrants and non-migrants from analyses of longitudinal population-based data is more mixed. We updated findings from review papers about migration and HIV risk in sub-Saharan Africa. We identified additional studies in PubMed searches from Jan 1, 2013, to Aug 14, 2014, with the terms “HIV” and “migra*” or “mobility”, and “Africa”. We also searched PubMed and Google Scholar to identify recent reports from international agencies about priorities for HIV, internal migration, and population geographies.

**Implications of all the available evidence**

Population-based studies to measure HIV infection and sexual behaviour need to take into account the social, behavioural, and situational risk factors present in the multiple environments to which migrants, especially those with circular migration patterns, are exposed. Our findings also suggest that a need exists to assess whether national HIV treatment and prevention programmes are providing services suitable for diverse, mobile populations.

**Added value of this study**

The updated data from the Africa Centre for Health and Population Studies Demographic Surveillance System are particularly informative because this was the first demographic surveillance system established in a highly mobile population with a severe HIV epidemic, and in which characterisation of migration and mobility was central to the conceptual and data model. We identified a positive association between recent migration and increased HIV prevalence for male and female residents. In our study we explicitly discussed the challenges of defining migration and the associated HIV risk in a rural African population with high levels of circular migration.

To examine the complexities of the association between migration and potential HIV risk, we used longitudinal data collected in ACDIS to compare population-level differences in sexual HIV risk behaviours and HIV prevalence with a range of indicators of migration and living arrangements.

## Methods

### Study design and population

Since January, 2000, longitudinal demographic and health data have been collected for roughly 90 000 household members from 12 000 households in a predominately rural 438 km^2^ demographic surveillance area (DSA) within the uMkhanyakude district of northern KwaZulu-Natal, South Africa.^15,16^ The DSA consists of Zulu tribal land with scattered households and a formal municipal township. Almost all the population speaks Zulu. The main sources of income for most households are formal employment and government grants, including pensions.^17^ Migrants move from the study area for various reasons, often related to employment or education, or to join partners or parents. Non-resident household members maintain social and physical connections with rural households through return visits and exchanges of material and physical support, including shared child care and financial support.^15^ Proportions of migrants are high, with 32% of women and 38% of men non-resident in 2008, and migration patterns differ by gender.^17,18^

The prevalence of HIV in this DSA has increased during the past decade and, in 2011, 29% of resident adults aged 15–49 years were infected with HIV.^19^ Crude HIV incidence in residents is estimated to be 2·63 infections per 100 person-years (95% CI 2·50–2·77).^20^ The local public HIV Treatment and Care Programme was initiated in 2004 and expanded rapidly. By December, 2011, 20 598 adults had initiated treatment, which is estimated to be 31% of all resident adults with HIV infection aged 15–50 years, thus contributing to the increasing HIV prevalence seen between 2005 and 2011.^19^ During the same period, no evidence suggested any increase in sexual risk taking behaviour. Condom use during most recent sexual intercourse with a regular partner increased significantly for men by an average of 2·6% (95% CI 1·5–3·7%) points per year and 4·1% (3·0–5·3) per year for women. Condom use at most recent sexual intercourse with a casual partner did not increase over time; it was more than 50% in 2005 compared with less than 30% with regular partners for both men and women.^21^

### Procedures

Demographic data (eg, births, deaths, and marriages) and information such as periods of absence and presence and migration events for all child and adult household members were collected twice per year from 2005 to 2011.^16^ Household membership was defined by key respondents and mainly related to perceptions of social connectedness and belonging. A household member was deemed a resident if they usually kept their day-to-day belongings and slept at the homestead; thus adults who are resident in the study area can be both inmigrants and people who have been residentially stable (always resident). Similarly, non-resident members included people who outmigrated from the study area and people who always lived in a place outside the study area.

Annual health surveys have been administered in the DSA since 2005, including collection of an anonymised blood sample for HIV testing and information about up to three most recent sexual partnerships in the past year.^16,21^ For the individual surveillance in each year, resident and non-resident eligibility lists were based on information from the household census in December of the previous year. All resident household members aged 15 years and older were eligible to participate, together with a stratified sample of non-resident household members (women aged 15–49 years and men aged 15–54 years). Sampling was stratified by sex and pattern of return visits to their household in the DSA, based on historically typical circular migration patterns (eg, monthly or annual return visits). Additionally, any non-resident individuals with a negative HIV test result in the HIV surveillance in the 2 years before the survey who were not selected in the current random sample were included in the non-resident sample. A special tracking fieldwork team established contact by telephone and arranged to interview non-residents at their home in the surveillance area during a return visit, or in their place of residence if outside the surveillance area. Tracking teams travelled as far as Gauteng province but not outside the country. The same questionnaires were used for resident and non-resident participants, and a dried blood spot sample obtained via fingerprick was collected from all consenting participants for HIV testing in a central laboratory.

The Nelson Mandela Medical School Research Ethics Committee of the University of KwaZulu-Natal (Durban, South Africa) gave ethics approval for all surveillance data collection activities.

### Statistical analysis

We identified four conceptually distinct indicators relating to participants' residential status at the time of each HIV survey and recent experiences of migration (ie, residential change): current residential status (ie, resident [usually sleeps and keeps their day-to-day belongings at the homestead] *vs* non-resident household members); recent mobility, based solely on the number of nights spent in the homestead during the past 6 months (at home every night, at home most nights [ie, less than ten nights away], or more than ten nights away); recent migration (migration to a homestead in the DSA at least once within the past 2 years); and migration type (in recent migrants, whether the migration was into or out of the surveillance area versus from another homestead in the DSA).

We examined the associations between the four indicators in each survey round using the corr function in Stata 13 to calculate the product-moment correlation coefficient. We used logistic regression models adjusted for differences in age composition and survey year to assess differences with respect to each migration indicator for HIV prevalence and sexual behaviour indicators. We used seven indicators of sexual behaviour: proportion of participants reporting they ever had sex, proportion of participants who were sexually active in the past year, proportion of participants reporting multiple partnerships in the past year, proportion of participants reporting a casual partnership, point-prevalence of concurrent sexual partnerships,^22^ proportion of participants reporting condom use at most recent sexual intercourse with the most recent regular partner, and proportion of participants reporting condom use at most recent sexual intercourse with the most recent casual partner. We analysed the data separately for men aged 17–54 years and women aged 17–49 years. We selected the minimum age limit of 17 years because sexual behaviour data were not available for 15–16 year olds in 2009 and 2011. We used Wald tests to assess statistical significance. Finally, in view of the weak associations between some migration indicators, we examined the risk of sexual HIV risk behaviours and HIV prevalence with respect to migration indicators in residents and non-residents separately.

We adjusted estimates for study non-participation and non-response with a previously described approach.^21^ Briefly, we made adjustments for survey non-participation by use of inverse-probability weights in strata defined by year, sex, age group, residence location (rural, periurban, urban, or non-resident). We used multiple imputation to adjust for missing responses.^21,23^

We analysed the data with the statistical software R 3.0.2. We implemented multiple imputation by creating customised imputation models for the imputation framework in the R package *mi*.^24^

### Role of the funding source

The funder of the study had no role in study design, data collection, data analysis, data interpretation, or writing of the report. The corresponding author had full access to all the data in the study and had final responsibility for the decision to submit for publication.

## Results

Between Jan 1, 2005, and Dec 31, 2011, many more eligible residents were contacted than were non-residents in all survey years (table 1). In both groups, contact generally decreased in the latter years of the survey. Differences in survey participation between residents and non-residents who were contacted were small, with no consistent trend across the years. Our ability to track and contact people differed between male and female non-residents (appendix). An increasingly large group of non-residents had return patterns (other return patterns) that were unpredictable or did not fall into the circular migration patterns that were common in the past. In any year, roughly a fifth of residents had migrated at least once in the past 2 years (table 2).

Current residential status was strongly associated with the number of nights spent in the DSA household in the past 6 months, but poorly associated with the indicators of recent migration and migration type. For women, the association between recent migration and residential status ranged from 0·13 to 0·35 during the study period (adjusted for sampling and response weights); association between the indicator of recent inmigration or outmigration and residential status ranged from 0·13 to 0·34; and association between the number of nights spent in the DSA household in the past 6 months and residential status ranged from −0·80 to −0·93. The pattern for men was similar: association between recent migration and residential status ranged from 0·07 to 0·29; association between the indicator of recent inmigration or outmigration and residential status ranged from 0·09 to 0·30; and association between the number of nights spent in the DSA household in the past 6 months and residential status ranged from −0·82 to −0·95.

When we adjusted for age and survey year, sexual risk behaviours were generally substantially higher in non-residents than in residents (table 3). This difference existed in both men and women, with some exceptions. The proportion of women who had a casual partner in the past year was significantly higher (p=0·0080) in non-residents than in residents, whereas the proportion of men who had casual partners did not differ between residential status groups. Condom use with regular partners was significantly higher in non-resident women than in resident women (p=0·0061), but the pattern was reversed in men (p=0·0047). Results for groups defined by the number of nights slept outside the DSA household in the past 6 months (recent mobility) were very similar to those for groups defined by current residential status (table 3). In women, results for groups defined by recent migration history were similar to those for groups defined by current residential status; whereas in men, significant differences only existed in comparisons of residential status and recent mobility, and not in comparisons of recent migration history. For both men and women, sexual risk behaviour was not significantly different when we compared recent migrants who migrated internally (ie, within the DSA) with those who migrated externally (ie, into or out of the DSA). HIV prevalence was not significantly different for any of the indicators.

In residents, differences existed with respect to migration indicators. Residents who had recently migrated and those who had spent more than ten nights away from their DSA household in the past 6 months had increased sexual risk behaviours, although most of the odds ratio estimates were not significant (table 4). Furthermore, HIV prevalence was significantly higher in male and female residents who had recently migrated than in residents who had not migrated in the past 2 years, and our data suggest that HIV prevalence is higher in recently mobile (spent more than ten nights away from DSA household in past 6 months) resident women than in resident women who were not recently mobile. In non-residents, no comparisons were significant, and in some cases confidence intervals were very wide.

## Discussion

Our results show that the previously identified increased levels of sexual risk behaviours in non-residents and those who spend few nights in their household in this study population^18,25,26^ have persisted in the post-ART rollout period, 2005–11. However, when we assessed indicators of recent migration (eg, migration over the past 2 years) irrespective of residential status, we identified no differences in sexual risk behaviour patterns for men, although we still detected differences in some sexual risk behaviours for women. These gender differences, combined with previous findings of differential patterns of migration by gender in this study population^25^ suggest that it might be necessary to design interventions for migrant men and women separately.^25^ Camlin and colleagues,^25^ noted that women were less likely to be non-resident members of rural households and more likely to migrate within the study area (internally) than were men.^25^

We noted that HIV prevalence in the population was not strongly related to any of our migration indicators (irrespective of residential status) or to residential status (regardless of migration history) in the post-ART era. However, when we examined HIV prevalence with respect to both migration and residential status, we saw that male and female residents who had migrated recently had higher HIV prevalence than did residents who had not migrated recently. This finding might be because HIV status depends on past risk behaviour and residential status is not strongly associated with migration history. Alternatively, this finding might result from the return of former non-residents to the study area upon ill-health or loss of employment or to access the local ART programme, or from a complex interplay between migration and local factors.^27,28^ Therefore, some residents might have acquired HIV while they were non-residents. This fact is crucial to discussions of targeted intervention strategies based on the identification of transmission hot spots,^29^ since the findings of previous studies showed localised spatial clustering of new HIV infections.^30,31^ Incident infections are those that occur in individuals who previously tested negative for HIV, but test positive for HIV at a later date. Dependent on the period of time between HIV tests, especially in a highly mobile population, not all incident cases identified in a transmission hot spot would necessarily have been acquired in that geographical location.

Residential status and indicators of recent migration and type of recent migration were poorly related to each other. We detected a strong, negative correlation between residential status and recent mobility, but this association varied between rounds, which might partly be explained by diverse domestic arrangements: even among the residents of the study area, roughly half of residents were reported not to sleep at home every night. These findings emphasise the importance and difficulties of identification of relevant migration indicators for HIV risk in this context and support recent suggestions from other researchers that knowledge about migration and HIV risk is incomplete.^2^ Efforts to appropriately conceptualise and measure migration in populations with generalised epidemics are timely in view of global initiatives that focus on migrants as one of the key populations with increased susceptibility to HIV.^32^ We propose that the emphasis on, and targeting of, labour migrants, who are often men, in HIV studies has tended to prioritise some migration types and flows, such as international and long-distance labour migration with infrequent return visits. By contrast, migration and mobility for many other people are poorly represented by the way that data are collected and analysed. For example, the increasing size of the subgroup of non-residents with return patterns classified as other during the period of this study (appendix), which restricts understanding of a much broader set of processes linked to residential instability.^33,34^ Our investigation of sexual risk behaviours and HIV prevalence in population subgroups defined by both migration indicators and residential status is a first step in development of a classification system of migration and mobility that further helps to identify individuals at risk of HIV. Our work supports the value of methodological innovations in the design of household-based studies, especially the use of a definition of household membership that includes resident and non-resident members. Such approaches could be extended further to collect information about the so-called floating population in an area (ie, household visitors, and household residents who are absent for short periods). Updated information about the effect of migration on migrants' access to, and engagement with, HIV testing and HIV care and treatment is also urgently needed.

HIV prevalence estimates from open population cohort studies show changes in population factors (eg, migration, deaths, and ageing), survey coverage, and HIV incidence.^3^ A strength of our study was our use of multiple imputation methods to try to ensure that the apparent changes were not artifacts of changes in the composition of respondents over time. In the multiple imputation, we used a wide range of variables collected as part of the demographic surveillance. However, selection might still have contributed to the results. The fact that we were only able to examine HIV prevalence rather than incidence is also a limitation, as was the fact that we had limited power to fully explore the different migration indicators for non-residents. From 2012, non-residents were no longer included in the Africa Centre individual surveillance and thus sexual behaviour or HIV data for this group were not available beyond 2011. However, no substantial changes have occurred in migration patterns since 2011 that would alter our interpretation of this study.

Our findings suggest a need to reassess whether the types of migration investigated as risk factors in the early studies of the HIV epidemic are still the most important in the ART era. Quantitative and qualitative studies and surveys to describe HIV infection and sexual behaviour in migrants, especially those with circular migration patterns, should examine the social, behavioural, and situational risk factors present in the multiple environments to which migrants are exposed (eg, both origin and destination communities). Such research is needed to inform HIV treatment and prevention programmes, and local information about migrants and highly mobile individuals will be crucial to the success of any place-based strategies to direct resources to transmission hotspots. Additionally, in HIV prevention approaches, including treatment as prevention, in which people with HIV infection are increasingly initiated on treatment early in the course of their infection, thought should be given to the widespread migration of this population. Thus, development of research and intervention strategies should proceed with caution unless the local context of migration and mobility is understood and can be incorporated.

## Figures and Tables

**Table 1:** Survey participation by year

		**Women (aged 17–49 years)**							**Men (aged 17–54 years)**						
		2005	2006	2007	2008	2009	2010	2011	2005	2006	2007	2008	2009	2010	2011
Eligible population*		21 129	21 234	22 011	22 289	21 849	22 726	22 267	20 399	20 586	21 344	21 656	21 382	22 100	21 779
Residents															
	Eligible	13 663	13 606	14 132	14 242	13 967	14 853	13 862	10 863	10 789	11 169	11 250	11 091	11 811	11 293
	Contacted†	10 784 (94%)	10 973 (98%)	10 722 (94%)	11 034 (96%)	10362 (95%)	10 194 (87%)	9515 (85%)	7965 (93%)	7782 (94%)	7302 (87%)	7968 (93%)	7406 (90%)	7101 (80%)	6858 (77%)
	Participated	7711 (60%)	7450 (56%)	6181 (47%)	5539 (41%)	5806 (44%)	5769 (45%)	5897 (50%)	4793 (47%)	4586 (45%)	3261 (34%)	3118 (30%)	2937 (29%)	3152 (33%)	3493 (40%)
	Complete sexual behaviour data‡	7403 (96%)	6892 (93%)	5429 (88%)	4359 (79%)	4403 (76%)	3834 (66%)	3608 (61%)	4527 (94%)	4249 (93%)	2800 (86%)	2434 (78%)	1988 (68%)	2113 (67%)	2203 (63%)
	HIV test result§	4422 (57%)	4104 (55%)	3638 (59%)	3570 (64%)	3371 (58%)	4299 (75%)	3920 (66%)	2763 (58%)	2424 (53%)	1851 (57%)	1974 (63%)	1802 (61%)	1987 (63%)	2013 (58%)
All non-residents															
	Eligible	7466	7628	7879	8047	7882	7873	8405	9536	9797	10175	10406	10291	10289	10486
	Sampled	1323 (18%)	1195 (16%)	1113 (14%)	1619 (20%)	1376 (17%)	1465 (19%)	1532 (18%)	1376 (14%)	1250 (13%)	1237 (12%)	1747 (17%)	1382 (13%)	1533 (15%)	1568 (15%)
	Contacted¶	715 (56%)	573 (57%)	544 (66%)	926 (74%)	747 (73%)	749 (53%)	643 (43%)	692 (52%)	539 (53%)	520 (59%)	853 (67%)	660 (66%)	777 (53%)	599 (39%)
	Participated	422 (57%)	324 (48%)	330 (45%)	424 (35%)	476 (47%)	412 (53%)	461 (70%)	355 (50%)	284 (43%)	325 (45%)	322 (28%)	406 (45%)	405 (50%)	438 (72%)
	Complete sexual behaviour data‡	392 (93%)	304 (94%)	306 (93%)	388 (92%)	438 (92%)	364 (88%)	399 (87%)	329 (93%)	272 (96%)	300 (92%)	298 (93%)	359 (88%)	359 (89%)	374 (85%)
	HIV test result§	299 (71%)	196 (60%)	245 (74%)	309 (73%)	362 (76%)	328 (80%)	325 (70%)	260 (73%)	192 (68%)	264 (81%)	255 (79%)	317 (78%)	346 (85%)	318 (73%)

Data are n or n (%).

* Resident and non-resident eligibility lists for the HIV surveillance were generated from a snapshot of the ACDIS database produced at the end of the previous year.

† By the time of the scheduled survey visit, on average 22% of eligible resident men and 18% of eligible resident women had died, outmigrated (thus making them no longer eligible for the resident sample), migrated to an unknown destination, or were unable to complete the survey for other reasons; all other individuals on the eligibility list were deemed contactable and contribute to the denominator for the contact rate.

‡ Number and percentage of participants who answered all sexual behaviour questions.

§ Number and percentage of participants who agreed to an HIV test.

¶ By the time of the scheduled survey visit, on average 15% of sampled non-resident men and 14% of sampled non-resident women were uncontactable, had died, or were unable to complete the survey for other reasons; all other individuals on the eligibility list were deemed contactable and contributed to the denominator for the contact rate.

**Table 2:** Indicators of recent experience of migration

		**Women**							**Men**						
		2005	2006	2007	2008	2009	2010	2011	2005	2006	2007	2008	2009	2010	2011
**Residents**															
Migrated at least once in past 2 years		20%	18%	20%	20%	19%	24%	21%	19%	19%	20%	21%	20%	25%	22%
	External migration events*	56%	61%	61%	58%	60%	57%	64%	63%	71%	68%	65%	71%	68%	73%
Spent <10 nights away from DSA household in past 6 months		95%	96%	95%	97%	98%	98%	98%	97%	96%	96%	98%	97%	98%	98%
**Non-residents**															
Migrated at least once in past 2 years		44%	45%	31%	45%	47%	38%	41%	39%	30%	31%	26%	44%	32%	32%
	External migration events*	65%	74%	69%	90%	71%	85%	86%	69%	81%	95%	64%	87%	96%	96%
Spent <10 nights away from DSA household in past 6 months		17%	5%	6%	4%	3%	4%	7%	15%	3%	1%	5%	4%	1%	3%

All percentage estimates are adjusted for sampling and response weights. Absolute numbers are not shown because of the stratified sample design for non-residents. The overall population size for each indicator is shown in table 1. DSA=demographic surveillance area.

* We defined external migration as the migration of an individual or household within the DSA to a household outside the DSA; the proportion reported represents external migration in individuals who migrated at least once in the past 2 years (recent migrants).

**Table 3:** Comparisons of sexual behaviour outcomes and HIV prevalence by migration indicators

	**Women**				**Men**			
	Non-residents *vs* residents (n=19 020)	Spent >10 nights away *vs* spent <10 nights away from DSA household in past 6 months (n=19 020)	Migrated at least once in past 2 years *vs* did not migrate (n=19 020)	External migration *vs* internal migration (n=7301)*	Non-residents *vs* residents (n=14 031)	Spent >10 nights away *vs* <10 nights away from DSA household in past 6 months (n=14 031)	Migrated at least once in past 2 years *vs* did not migrate (n=14 031)	External migration *vs* internal migration (n=4681)*
Sexually active in past 12 months	1·19 (0·96–1·49)	1·24 (1·00–1·54)	1·22 (1·03–1·45)†	0·94 (0·69–1·29)	1·53 (1·14–2·05)†	1·55 (1·15–2·08)†	1·12 (0·84–1·49)	1·64 (0·94–2·87)
Multiple partners in past year	3·10 (1·88–5·12)†	2·59 (1·53–4·40)†	2·04 (1·07–3·89)†	0·90 (0·38–2·12)	1·66 (1·32–2·09)†	1·65 (1·31–2·09)†	0·97 (0·73–1·30)	1·65 (0·89–3·06)
Point prevalence of concurrency	3·90 (1·64–9·25)†	3·34 (1·35–8·25)†	2·53 (0·85–7·49)	2·35 (0·38–14·61)	1·80 (1·34–2·43)†	1·82 (1·35–2·46)†	0·87 (0·59–1·28)	1·41 (0·64–3·10)
Had a casual partner in the past year	1·69 (1·15–2·50)†	1·55 (1·04–2·32)†	1·55 (1·03–2·33)†	0·85 (0·40–1·79)	1·11 (0·86–1·44)	1·10 (0·85–1·43)	0·93 (0·69–1·24)	1·25 (0·63–2·51)
Condom use at most recent sexual intercourse with casual partner	2·06 (0·94–4·52)	1·87 (0·86–4·06)	1·69 (0·82–3·50)	0·33 (0·00–38·56)	1·18 (0·70–1·98)	1·12 (0·69–1·82)	0·74 (0·45–1·23)	0·82 (0·36–1·85)
Condom use at most recent sexual intercourse with regular partner	1·38 (1·10–1·73)†	1·42 (1·13–1·77)†	1·19 (0·99–1·42)	1·16 (0·86–1·57)	0·70 (0·55–0·90)†	0·70 (0·54–0·89)†	0·90 (0·66–1·22)	0·84 (0·48–1·47)
HIV prevalence	0·77 (0·55–1·06)	0·85 (0·64–1·14)	1·07 (0·90–1·27)	0·96 (0·70–1·32)	0·77 (0·55–1·10)	0·79 (0·58–1·09)	0·92 (0·68–1·24)	1·02 (0·53–1·96)

Data are adjusted odds ratio (95% CI), adjusted for linear trend over time and 5 year age groups. Women were aged 17–49 years and men were aged 17–54 years. DSA=demographic surveillance area.

* In individuals who migrated at least once in the past 2 years (recent migrants).

† p<0·05.

**Table 4:** Comparisons of sexual behaviour outcomes and HIV prevalence by migration groups for residents and non-residents

	**Women who migrated at least once in past 2 years *vs* women who did not migrate**		**Women who spent >10 nights away *vs* women who spent <10 nights away from DSA household in past 6 months**		**Men who migrated at least once in past 2 years *vs* men who did not migrate**		**Men who spent >10 nights away *vs* men who spent <10 nights away from DSA household in past 6 months**	
	Residents (n=17 895)	Non-residents (n=2203)	Residents (n=17 895)	Non-residents (n=2203)	Residents (n=12 921)	Non-residents (n=1875)	Residents (n=12 921)	Non-residents (n=1875)
Sexually active in the past 12 months	1·17 (1·09–1·26)*	1·26 (0·83–1·92)	1·20 (1·02–1·41)*	1·44 (0·79–2·62)	1·20 (1·09–1·32)*	0·98 (0·51–1·89)	1·36 (1·08–1·72)*	1·16 (0·53–2·54)
Multiple partners in the past year	1·27 (0·97–1·66)	1·70 (0·56–5·18)	1·80 (1·14–2·84)*	0·86 (0·23–3·14)	1·15 (1·03–1·30)*	0·76 (0·46–1·25)	1·21 (0·95–1·55)	1·21 (0·62–2·36)
Point prevalence of concurrency	1·26 (0·75–2·11)	1·99 (0·28–14·10)	2·28 (1·08–4·83)*	1·92 (0·06–62·20)	1·09 (0·94–1·27)	0·68 (0·36–1·29)	1·06 (0·76–1·48)	1·85 (0·73–4·72)
Had a casual partner in the past year	1·18 (0·99–1·40)	1·79 (0·82–3·91)	1·08 (0·76–1·53)	0·77 (0·27–2·22)	1·03 (0·92–1·15)	0·76 (0·45–1·30)	1·12 (0·41–3·06)	1·12 (0·41–3·06)
Condom use at most recent sexual intercourse with casual partner	1·08 (0·78–1·50)	1·56 (0·05–46·41)	1·04 (0·52–2·10)	1·02 (0·06–18·12)	0·90 (0·72–1·12)	0·60 (0·17–2·14)	1·32 (0·79–2·18)	1·12 (0·12–10·24)
Condom use at most recent sexual intercourse with regular partner	1·08 (1·00–1·16)*	1·15 (0·78–1·69)	1·12 (0·97–1·29)	1·75 (0·88–3·46)	0·94 (0·84–1·05)	0·88 (0·52–1·50)	0·77 (0·61–0·97)*	0·90 (0·35–2·35)
HIV prevalence	1·18 (1·10–1·26)*	1·06 (0·73–1·55)	1·16 (0·97–1·39)	1·45 (0·73–2·88)	1·19 (1·07–1·33)*	0·67 (0·31–1·49)	0·98 (0·75–1·29)	0·90 (0·38–2·11)

Data are adjusted odds ratio (95% CI), adjusted for linear trend over time and 5 year age group. Women were aged 17–49 years and men were aged 17–54 years. The indicator of external migration *vs* internal migration could only be assessed for resident men and women who migrated in the past 2 years because numbers were too small for non-residents; no estimates were statistically significant and data are not shown. DSA=demographic surveillance area.

* p<0·05.
