# Supplementary material for: Migration, sexual behaviour, and HIV risk: a general population cohort in rural South Africa
Source: Lancet HIV. 2015 Apr 16;2(6):e252–9. doi: 10.1016/S2352-3018(15)00045-4 (PMC4533230; doi:10.1016/S2352-3018(15)00045-4)
Supplement: Supplementary appendix [file mmc1.pdf]

THE LANCET HIV

Supplementary appendix

This appendix formed part of the original submission and has been peer reviewed. We post it as supplied by the authors.

Supplement to: McGrath N, Eaton JW, Newell M-L, Hosegood V. Migration, sexual behaviour, and HIV risk: a general population cohort in rural South Africa. *Lancet HIV* 2015; published online April 17. http://dx.doi.org/10.1016/S2352-3018(15)00045-4.

Supplemental table 1: Non-resident eligible population by return visit patterns, for each sex in each year

		**Women**							**Men**						
		**2005**	**2006**	**2007**	**2008**	**2009**	**2010**	**2011**	**2005**	**2006**	**2007**	**2008**	**2009**	**2010**	**2011**
**Among Non-Residents:**															
**Frequent return**	Eligible	1227	1151	1160	1019	789	573	701	1303	1259	1245	1075	775	648	678
	Sampled	152	189	125	190	186	186	199	145	198	123	188	182	190	199
	Sampled %	*12%*	*16%*	*11%*	*19%*	*24%*	*32%*	*28%*	*11%*	*16%*	*10%*	*17%*	*23%*	*29%*	*29%*
	Contacted %	*72%*	*54%*	*58%*	*58%*	*55%*	*56%*	*41%*	*72%*	*46%*	*54%*	*64%*	*50%*	*52%*	*35%*
	Participated	66	47	30	39	54	41	50	58	42	34	29	42	36	31
	Participated %	*64%*	*46%*	*41%*	*35%*	*52%*	*39%*	*61%*	*59%*	*46%*	*52%*	*24%*	*46%*	*36%*	*44%*
**Monthly return**	Eligible	977	879	743	619	541	488	439	1143	1065	920	744	651	597	523
	Sampled	172	181	152	192	185	200	205	169	223	173	195	184	201	205
	Sampled %	*18%*	*21%*	*20%*	*31%*	*34%*	*41%*	*47%*	*15%*	*21%*	*19%*	*26%*	*28%*	*34%*	*39%*
	Contacted %	*49%*	*50%*	*43%*	*58%*	*51%*	*46%*	*40%*	*47%*	*37%*	*38%*	*48%*	*46%*	*48%*	*35%*
	Participated	36	40	40	47	51	48	55	24	39	40	28	41	42	54
	Participated %	*45%*	*44%*	*61%*	*42%*	*54%*	*52%*	*68%*	*32%*	*47%*	*61%*	*30%*	*48%*	*44%*	*76%*
**Occasional return**	Eligible	1457	1466	1382	1247	1246	993	957	1754	1762	1582	1396	1502	1197	1071
	Sampled	152	172	154	173	157	189	196	162	149	167	178	153	191	200
	Sampled %	*10%*	*12%*	*11%*	*14%*	*13%*	*19%*	*20%*	*9%*	*8%*	*11%*	*13%*	*10%*	*16%*	*19%*
	Contacted %	*52%*	*42%*	*47%*	*58%*	*57%*	*52%*	*37%*	*44%*	*39%*	*43%*	*52%*	*42%*	*49%*	*30%*
	Participated	39	35	48	46	47	44	46	23	26	47	34	37	55	48
	Participated %	*52%*	*48%*	*66%*	*46%*	*52%*	*44%*	*64%*	*34%*	*45%*	*66%*	*37%*	*57%*	*59%*	*81%*
**Annual return**	Eligible	627	558	478	433	422	370	308	1399	1317	1142	1033	970	857	771
	Sampled	170	180	174	199	189	195	200	174	176	175	196	184	199	206
	Sampled %	*27%*	*32%*	*36%*	*46%*	*45%*	*53%*	*65%*	*12%*	*13%*	*15%*	*19%*	*19%*	*23%*	*27%*
	Contacted %	*46%*	*39%*	*41%*	*54%*	*49%*	*42%*	*42%*	*49%*	*43%*	*38%*	*43%*	*38%*	*50%*	*33%*
	Participated	35	44	38	51	64	48	61	29	36	42	39	44	52	49

	Participated %	*46%*	*62%*	*54%*	*48%*	*69%*	*59%*	*73%*	*35%*	*48%*	*64%*	*46%*	*63%*	*53%*	*71%*
**Other return pattern**[1]	Eligible	2666	3278	3687	4042	4381	4939	5469	3379	4079	4775	5349	5868	6429	6885
	Sampled	165	177	79	178	156	185	201	168	189	88	181	154	191	200
	Sampled %	*6%*	*5%*	*2%*	*4%*	*4%*	*4%*	*4%*	*5%*	*5%*	*2%*	*3%*	*3%*	*3%*	*3%*
	Contacted %	*46%*	*44%*	*58%*	*57%*	*40%*	*49%*	*42%*	*37%*	*42%*	*34%*	*46%*	*36%*	*40%*	*42%*
	Participated	33	41	27	35	32	54	56	26	40	12	15	27	45	63
	Participated %	*46%*	*53%*	*59%*	*34%*	*52%*	*59%*	*66%*	*44%*	*51%*	*40%*	*18%*	*49%*	*58%*	*76%*
**Had a negative HIV test result within surveillance in previous 2 yrs**	Eligible	512	296	429	687	503	510	531	558	315	511	809	525	561	558
	Contacted %	*62%*	*55%*	*51%*	*58%*	*61%*	*55%*	*45%*	*56%*	*50%*	*44%*	*48%*	*57%*	*56%*	*44%*
	Participated	213	117	147	206	228	177	193	195	101	150	177	215	175	193
	Participated %	*69%*	*72%*	*67%*	*52%*	*74%*	*63%*	*81%*	*64%*	*64%*	*67%*	*46%*	*72%*	*56%*	*78%*

[1] There is a large group of non-residents whose return patterns are unpredictable or do not fall into the circular migration patterns that were common in the past.
